# Design, Fabrication and Characterization of Pressure-Responsive Films Based on The Orientation Dependence of Plasmonic Properties of Ag@Au Nanoplates

**DOI:** 10.1038/s41598-017-01928-5

**Published:** 2017-05-10

**Authors:** Li-Shun Fu, Wen-Shou Wang, Cheng-Yan Xu, Yao Li, Liang Zhen

**Affiliations:** 1 School of Materials Science and Engineering, Harbin Institute of Technology, Harbin, 150001 China; 2 Department of Chemistry, University of California, Riverside, CA 92521 USA; 3 Kuang-Chi Institute of Advanced Technology, Shenzhen, 518057 China; 4 National Engineering Research Center for Colloidal Materials, Shandong University, Jinan, 250100 China; 5 School of Chemistry and Chemical Engineering, Shandong University, Jinan, 250100 China; 6 Center for Composite Materials, Harbin Institute of Technology, Harbin, 150001 China

## Abstract

A novel pressure-responsive polymer composite film was developed based on Ag@Au composite nanoplates (NPLs) and polyvinylpyrrolidone (PVP) by using Au nanoparticles as concentration reference. The orientation change of Ag@Au NPLs is impelled by the deformation of polymer matrix under pressure, resulting in its localized surface plasmon resonance (LSPR) intensity change of in-plane dipolar peak. The intensity ratio between plasmon peak of Au nanoparticles and in-plane dipolar peak of Ag@Au NPLs relies on the intensity and duration of pressure. By adjusting the viscosity of the polymer, the orientation change of LSPR may respond to a wide range of stresses. This pressure sensitive film can be utilized to record the magnitude and distribution of pressure between two contacting surfaces via optical information.

## Introduction

Research in noble metal nanoparticles with localized surface plasmon resonance (LSPR) properties has been around for a long time due to their broad applications in sensing^1–3^, catalysis^4–6^, therapeutics^7–9^, optical nanoantennas^10, 11^, and surface-enhanced Raman spectroscopy^12–15^. Their applications in the field of sensing focus on biosensors and chemical sensors based on the change of plasmonic properties related to refractive-index^1, 16, 17^, morphology^18, 19^, and assembly^3, 20–22^. It is noteworthy that the orientation of noble metal nanoparticles such as Au nanorods (NRs) could change with the deformation of polymer matrix, as well as their LSPR properties. For example, Liz-Marzán and co-workers obtained uniform distributions of aligned AuNRs by stretching AuNR-poly(vinyl alcohol) composite films^23^. The selective excitation of transverse and longitudinal mode of such oriented films can be achieved using polarized light. Agarwal *et al*. realized the reversible alignment of AuNRs in mechano-responsive elastomers^24^. The angle of stretching direction and incident light polarization had obvious influence on the absorption of longitudinal mode of AuNRs. We previously reported a new prototype of pressure sensor founded in the orientation dependence of LSPR properties of AuNRs^25^. The intensity ratio of resonance peaks of LSPR, which changes with the orientation of nanostructures impelled by the deformation of polymer matrix under pressure, can be used as a pressure indicator. However, the intensity of transverse resonance of AuNRs is much smaller than that of longitudinal mode, and in practical applications it is difficult to measure its small variation, which limits the measurement range of pressure. Blended noble metal nanostructures with various plasmon peaks have different pressure-sensitive LSPR properties, providing an alternative strategy to overcome the drawbacks of AuNRs to extend the measurement range of pressure.

## Results

**Figure 1:**
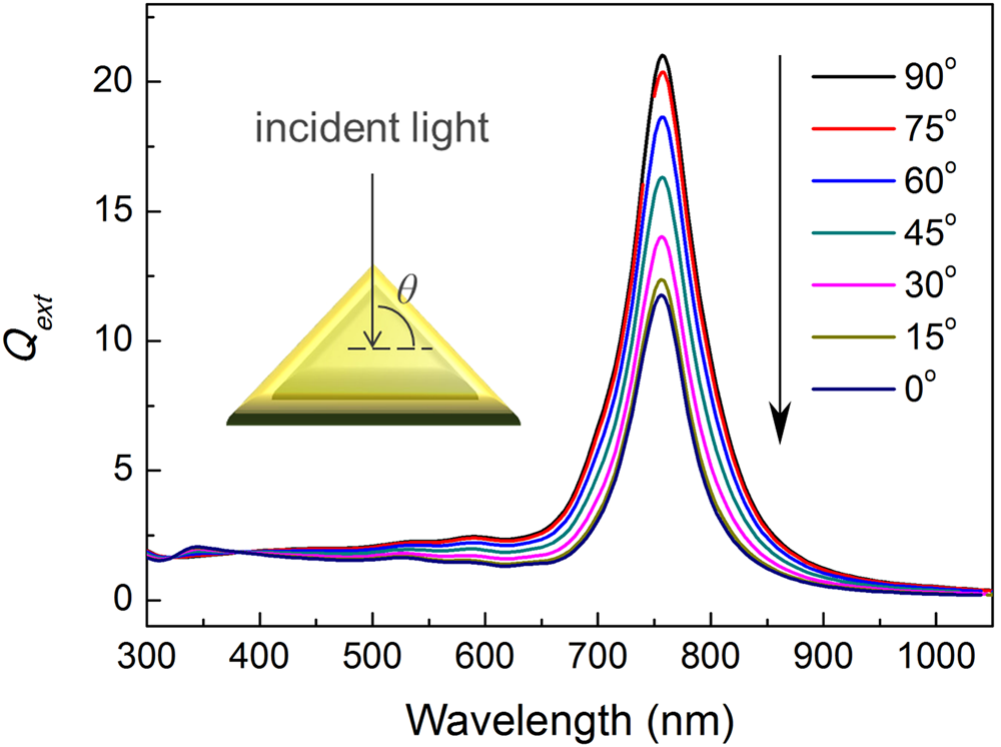
DDA simulation of extinction efficiency spectra of individual Ag@Au NPL (triangular prism, edge length: 90 nm, thickness: 20 nm, Au shell thickness: 4 nm, 8790 dipoles were utilized in the simulation) in various directions under unpolarized light.

Here, we reported a polymer composite film based on Ag@Au composite nanoplates (NPLs) and polyvinylpyrrolidone (PVP) by using Au nanoparticles as concentration reference. AgNPLs with tunable size and uniform shape can be synthesized via seeded growth method^26–29^, however, the stability of AgNPLs in the polymer matrix is a challenging due to oxidation by surfactant-assisted etching. Ag@Au NPLs obtained by epitaxial growth of a layer of gold on silver can enhance their stability. Figure 1 shows simulated extinction spectra of a Ag@Au NPL with its direction changing from parallel to perpendicular to the incident light, which is acquired from discrete dipole approximation (DDA)^30, 31^. The main peaks in the spectra are in-plane dipolar modes^32^. It can be seen that the intensity of the in-plane dipolar resonance of Ag@Au NPL decreases monotonically when its orientation angle changes from 90° to 0° (the direction of incident light is set as 0°). Thus, the in-plane dipolar resonance of Ag@Au NPL can be used for construction of new system based on the orientation change. As for Au nanoparticles (NPs), the plasmon peak is not sensitive to the orientation change and not overlapped with that of AgNPLs so that AuNPs can be utilized as a reference.

In this work, Ag@Au NPLs and AuNPs are homogenously embedded in the polymer matrix. Because the intensity of in-plane dipolar peak of Ag@Au NPLs is related to the orientation and area concentration while that of plasmon peak of AuNPs is related to the area concentration, the intensity ratio between these two peaks is related to the orientation of Ag@Au NPLs. The working mechanism is illustrated in Figure S1(a). Ag@Au NPLs are randomly oriented in the composite film before experiencing the pressure. When an external force is applied on the composite film, polymer flow will appear in the plane perpendicular to the applied pressure and in this polymer flow field Ag@Au NPLs will change their orientations. The intensity ratio between these two plasmon peaks will change due to the realignment of Ag@Au NPLs to a more ordered condition. The realignment behavior of Ag@Au NPLs embedded in the polymer matrix is similar to that of AuNRs^25^. From the cross section view, planar extensional flow will be induced in the composite film when an external force F is perpendicularly applied on the plain of the film, as shown in Figure S1(b). The horizontal portion of the flow velocity will rise with the rise in the length from the middle point of the applied pressure, while the vertical portion will decline with the rise of the deepness of composite film. The suspended NPLs will show rotational and translational motions under this velocity gradient. We could just take the direction change of NPLs relative to *z*-axis into account because the orientation related change of LSPR of Ag@Au NPLs only depends on the relative angle between the base plane of NPL and the incident direction of the light (The incident light is parallel to the external force).

Considering that rod-like and plate-like particles can be regarded as prolate and oblate spheroid, respectively, the rotation behavior of these two shapes can be treated using the same method. So the rotational motion for plate-like particles in the planar extensional flow ($\partial u/\partial \rho =\mathop{\gamma }\limits^{\cdot }/2$, $\partial u/\partial z=-\,\mathop{\gamma }\limits^{\cdot }$, where *u* is the velocity of fluid, $\mathop{\gamma }\limits^{\cdot }$ is the deformation rate in the direction of applied pressure, as shown in Figure S1(c)) can also be described by the equation used for rod-like particles derived in previous work^25^:

1 $$\begin{array}{ccc}\tan \,\theta  & = & \tan \,{\theta }_{0}\cdot \exp (\frac{3}{2}\frac{{r}_{e}^{2}-1}{{r}_{e}^{2}+1}\gamma )\end{array}$$

where *θ* is the orientation angle in the *ρz* plane of an individual particle at time *t*, *θ*_*0*_ is the orientation angle at the beginning (0 s), *r*_*e*_ is the aspect ratio of ‘equivalent ellipsoidal’ of plates, *γ* is the deformation of the film in the thickness direction. Therefore, Ag@Au NPLs that are randomly distributed in the polymer will become more parallel to the base plane of the polymer film. So the intensity of in-plane dipolar mode of AgNPLs should increase after experiencing the external force if the concentration change of Ag@Au NPLs can be neglected. Actually, the area concentration of Ag@Au NPLs will decrease because composite films become thinner during compression, resulting in the decrease of the intensity of plasmon peaks. The plasmon peak of another nanostructure can be used for concentration calibrator which could eliminate the concentration-dependent intensity change of in-plane dipolar mode of Ag@Au NPLs. Au nanoparticle (NP) is a good choice of concentration calibrator because its resonance peak is located at about 520 nm and the plasmon intensity is independent of the orientation change. So it is believed that the ratio of the intensity of two plasmon peaks (*I*_*Ag*_ */I*_*Au*_) should rise after the external force is applied on the composite film, and this change can be utilized to express the pressure experienced by the films.

**Figure 2:**
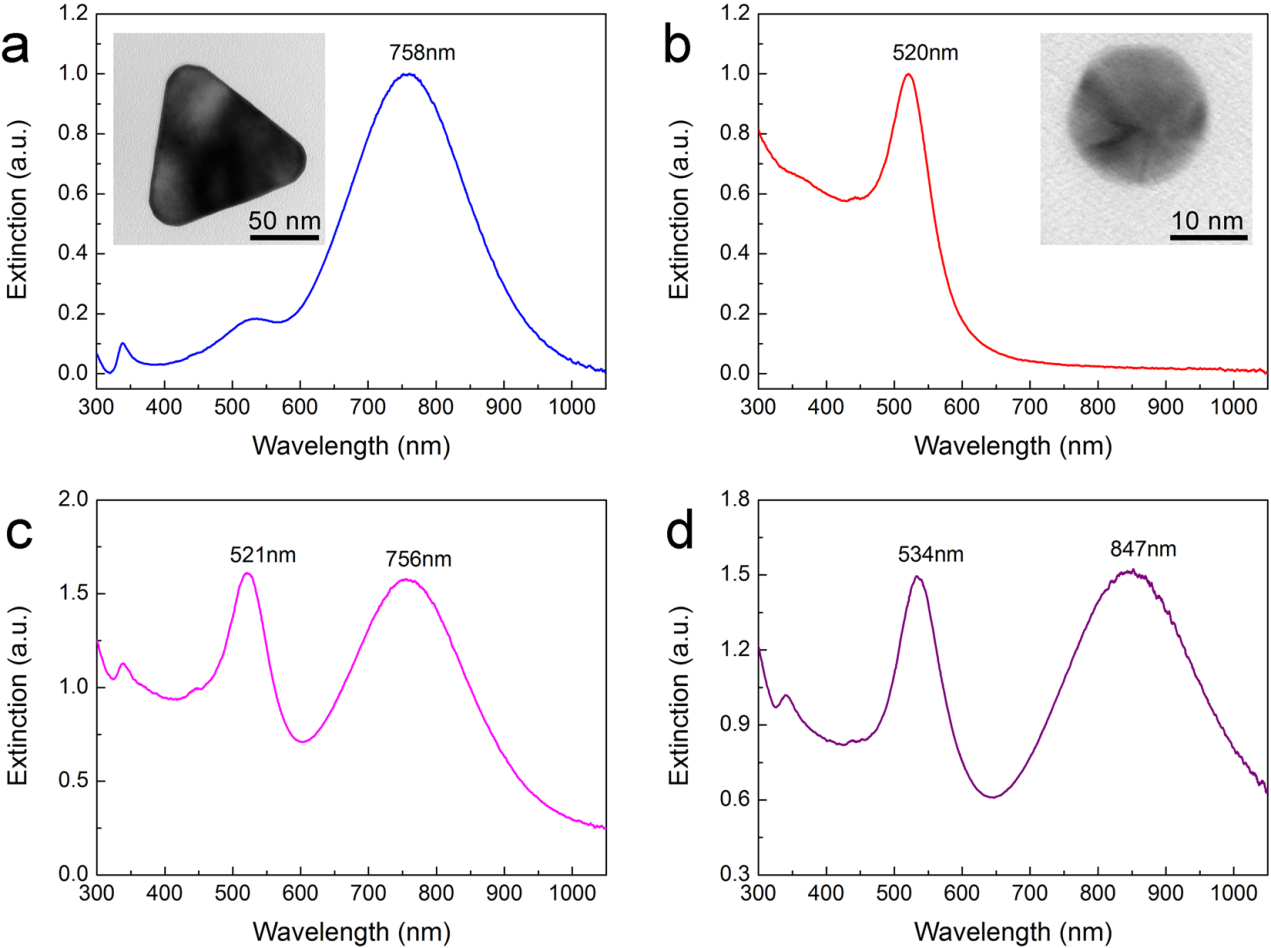
(**a**) Extinction spectra of Ag@Au NPLs. The inset is a representative TEM image of Ag@Au NPLs. (**b**) Extinction spectra of AuNPs. The inset is its corresponding TEM image. (**c**) Extinction spectra of Ag@Au NPLs and AuNPs in water. (**d**) Extinction spectra of Ag@Au NPLs and AuNPs in polymer composite films.

To fabricate pressure sensitive composite films, AgNPLs were first prepared via a seeded growth method^26^. After epitaxial growth^33^, triangular Ag@Au NPLs were obtained and the in-plane dipolar peak is located at 758 nm, as shown in Fig. 2(a). Then the concentration calibrator AuNPs were prepared by a slightly modified Turkevich method^34, 35^, and corresponding plasmon peak is located at 520 nm (Fig. 2(b)). To evaluate the compatibility, we mixed Ag@Au NPLs and AuNPs in aqueous solution. It can be seen in Fig. 2(c) that both the plasmon peaks of Ag@Au NPLs and AuNPs did not change drastically, indicating that they both dispersed well in water after mixing. Then the mixture of Ag@Au NPLs and AuNPs were mixed with ethanol solution containing PVP (*M*_*w*_ = 360,000) and polyethylene glycol (PEG, *M*_*w*_ = 400) to produce a glutinous dispersion, which was utilized to form a composite film by solution casting after evaporation of the solvent. PVP was selected as the polymer matrix due to its low optical absorption in visible range, good forming ability of film, intense adhesion to the surface of gold, and excellent solubility in water and ethanol^36^. PEG as a good plasticizer which is fully mixable with PVP could change the fluidity of the composite film^36^. PDMS has been used as the polymer of films, however, Ag@Au NPLs are aggregated in this type of films because solvent transfer issues cannot be solved.

Compared with the mixture solution of Ag@Au NPLs and AuNPs, the polymer composite films show red shift of plasmon peaks (Fig. 2(d)) due to the rise in the refractive index of the polymer matrix (water: 1.333, PVP: 1.527, PEG: 1.467). Peak broadening is not found in the spectra, which indicates that Ag@Au NPLs and AuNPs were well dispersed in the polymer matrix without obvious aggregation^23, 25^. The concentration of Ag@Au NPLs and AuNPs per film area was about 0.06~0.07 and 0.05~0.06 mg/cm^2^, respectively, while the thickness of Ag@Au NPL-polymer film was about 350 µm. It need to be pointed out that it is difficult to fabricate thin films used for the experiments in this work, considering that Ag@Au NPLs and AuNPs need to be well dispersed in the polymer films composed of PVP and PEG and the polymer films with a large area with diameter of 22 mm need to be peeled off from the mold. The volume fraction of Ag@Au NPLs is about 0.02%, much smaller than *1/r* ^*2*^ (*r* is the aspect ratio of Ag@Au NPLs), which qualifies the composite film as a dilute suspension where nanoplates are capable of free rotation^37^. In fact, the size of Ag@Au NPLs is much smaller than their calculated average interparticle spacing so that Ag@Au NPLs are able to freely move and rotate, which makes it possible to take advantage of film deformation for pressure sensing.

**Figure 3:**
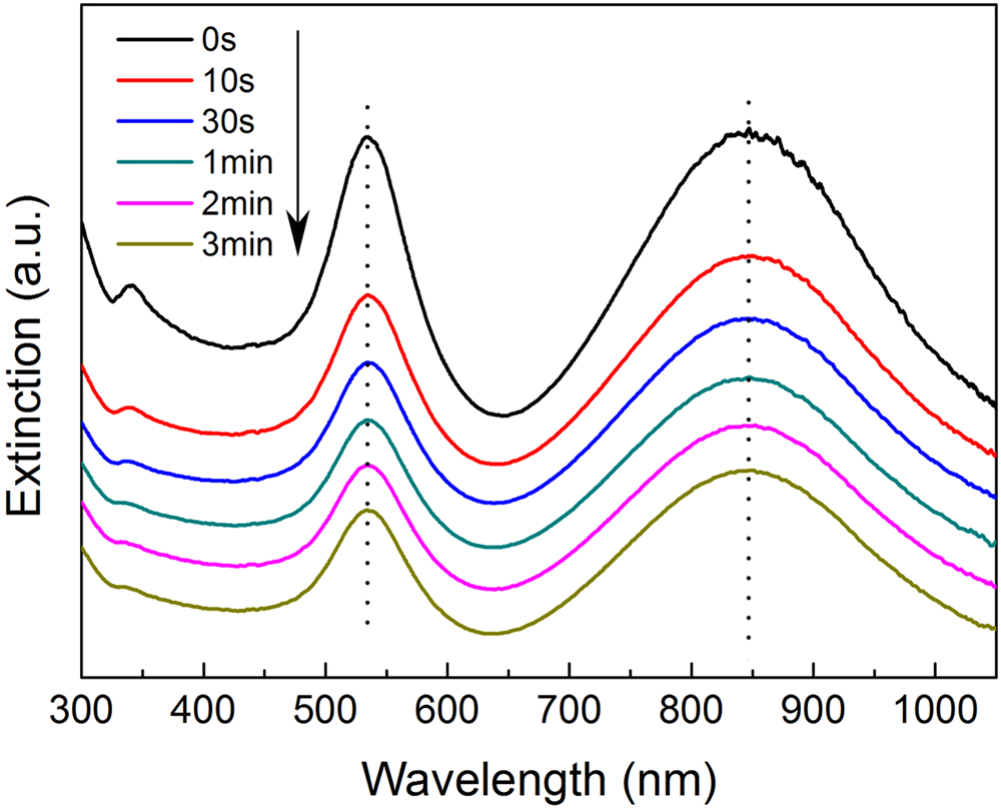
The absorbance spectra of Ag@Au NPLs and AuNPs in the composite film after experiencing a constant pressure for various duration. For clarity, the spectra are arbitrarily moved along the *y*-axis.

To appraise the pressure sensitivity of films, one piece of Ag@Au NPL-polymer film was first put on a fixed pressure (4 × 10^3^ psi) for 3 min. It is evident that the intensity of plasmon peak of AuNPs and in-plane dipolar peak of Ag@Au NPLs both declined during compression, as seen in Fig. 3. This is because that the area concentration of AuNPs and Ag@Au NPLs both decline with the increase of area and the decrease of thickness of composite film during the process of pressing. Moreover, the positions of two LSPR peaks did not vary during compression, which indicates no apparent variation in the shape of AuNPs and Ag@Au NPLs. However, the intensity of these two plasmon peaks did not vary as considerably as simulation. This can be ascribed to that DDA simulation results of extinction spectra are for individual AgNPL with a defined orientation, while actually there are numberless Ag@Au NPLs with different aspect ratios and orientations in the composite film. Furthermore, the orientation variation of AgNPLs in the polymer flow is restricted due to the deformation which is actually not obvious.

**Figure 4:**
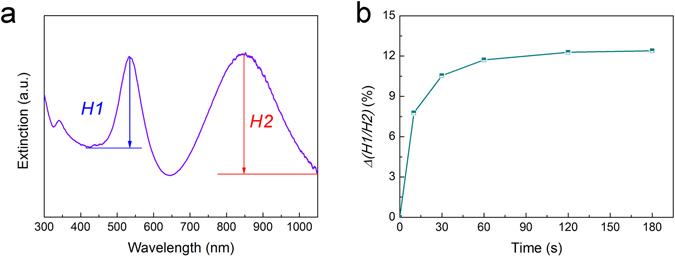
(**a**) Definition of the intensity of resonance peak of AuNPs and in-plane dipole resonance peak of Ag@Au NPLs. (**b**) Plot of *Δ(H1/H2)* for the composite film applied with a constant pressure for various duration originated from the spectra in Fig. 3.

If the changes of plasmon peak of AuNPs and in-plane dipolar peak of Ag@Au NPLs are analysized quantitatively, the proper baselines for the resonance bands need to be defined. For convenience, the left shoulder of the band is chosen as the baseline of plasmon peak of AuNPs while the right shoulder as the baseline of in-plane dipolar peak of Ag@Au NPLs, as indicated in Fig. 4(a). The intensity of plasmon peak of AuNPs and in-plane dipolar peak of Ag@Au NPLs was expressed as *H1* and *H2*, respectively. It need to be pointed out that the baseline for plasmon peak of AuNPs is much higher than that for in-plane dipolar peak of Ag@Au NPLs, which can be ascribed to that non-LSPR absorption from 5d electron transition in the spectra before 496 nm could not be counted a part of an LSPR absorption peak^38^.

As mentioned before, the intensity ratio of two LSPR peaks *H1/H2* is merely related to the orientation of Ag@Au NPLs. To correlate these small variations in the intensity of the plasmon peaks with the orientation change of the NPLs, *Δ(H1/H2)* is defined as follows:

2 $$\begin{array}{ccc}\Delta (H{1}/H{2}) & = & \frac{{(H1/H{2})}_{{0}}-{(H{1}/H{2})}_{t}}{{(H{1}/H{2})}_{{0}}}\end{array}$$

where *(H1/H2)*_*0*_ and *(H1/H2)*_*t*_ are the intensity ratios of two LSPR peaks at the very beginning (0 s) and time point *t*, respectively. Firstly, the obvious dependence of *Δ(H1/H2)* on the direction of the NPLs was confirmed taking advantage of the data of simulation. As seen from Figure S3, if the beginning angle of a Ag@Au NPL relative to the incident light is set as 45° and the NPL rotates a little, the intensity ratio of two modes vary considerably. For example, if the angle between the Ag@Au NPL and the incident light rises by 15°, *Δ(H1/H2)* could increase to 12.50%, indicating that this variable is suitable for investigating the plasmonic variations related with the small change of the direction of Ag@Au NPLs. We also confirm the effectiveness of this variable by treating spectra change of the composite film after the application of the pressure for different durations (Fig. 3). As indicated in Fig. 4(b), the corresponding *Δ(H1/H2)* values change more evidently than the extinction spectra themselves.

**Figure 5:**
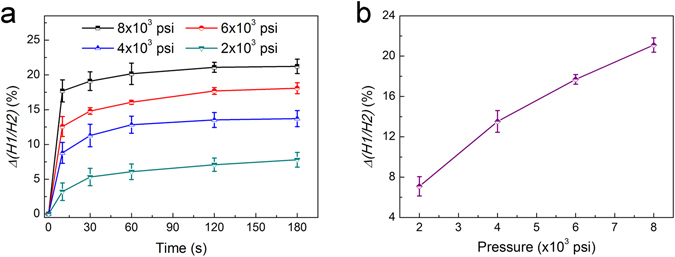
(**a**) Plots of *Δ*(*H1*/*H2*) for composite films applied with various pressures for different periods of time. (**b**) Plot of *Δ*(*H1*/*H2*) for composite films with 30 wt% PEG that experienced different pressures for 2 min.

From Equation (1), it can be inferred that the variation of orientation angle of Ag@Au NPLs will rise with the extension of the deformation of composite films. Burgers model, widely utilized to investigate the viscoelasticity of polymer, could provide the relationship of strain and stress. When the stress is constant, the deformation at time *t* could be described as^39^:

3 $$\gamma =\frac{{\sigma }_{0}}{{E}_{1}}+\frac{{\sigma }_{0}}{{E}_{2}}(1-{e}^{-t/\tau })+\frac{{\sigma }_{0}}{\eta }t$$

where *σ*_*0*_ is the applied stress at the beginning, *E*_*1*_ and *E*_*2*_ are the modulus of instantaneous elastic deformation and high elastic deformation, respectively, *η* is the viscosity of viscous flow, and τ is retardation time of high elastic deformation. So the deformation of polymer film can be expressed by three stages: the instantaneous elastic stage, the transition stage (the deformation rate declines), and the steady stage (the deformation rate varies very bit)^25^. From Equations (1) and (3), it can be concluded that the orientation angle of Ag@Au NPLs with the same aspect ratios will rise with the rise in the intensity and duration of applied stress on the composite films. As a result, the ratio between the plasmon peak of AuNPs and the in-plane dipolar peak of Ag@Au NPLs will decline, causing a variation of *Δ(H1/H2)*. To validate this assumption, various pressures (8 × 10^3^, 6 × 10^3^, 4 × 10^3^ and 2 × 10^3^ psi) were applied on a supply of composite films for the same range of time. During pressing, the variations of extinction spectra for different duration (10 s, 30 s, 1 min, 2 min and 3 min) were noted down. The relationship between *Δ(H1/H2)* and the pressure that the composite film experienced for different duration is plotted in Fig. 5(a). When the applied pressure remained constant, *Δ(H1/H2)* rose very fast in the beginning 10 s, then the rise speeded down, and stayed almost unchanged after 2 min. Based on the dependence of *Δ(H1/H2)* on the orientation change plotted in Figure S3, the orientation change of Ag@Au NPLs in the composite films with time can be obtained, as shown in Figure S4. It can be seen that the orientation angle of Ag@Au NPL first rose very fast, corresponding to the instantaneous elastic deformation; then the variation of orientation angle speeded down gradually, corresponding to the transition stage of deformation; at last the angle between the Ag@Au NPL and incident light varied very little, corresponding to the steady stage of deformation.

When the duration of applied pressure is fixed at 2 min and the proportion of PEG is 30 wt%, *Δ(H1/H2)* at pressure 2 × 10^3^, 4 × 10^3^, 6 × 10^3^ and 8 × 10^3^ psi is 7.09%, 13.52%, 17.69% and 21.09%, respectively, as seen in Fig. 5(b). As expected, under higher pressure the polymer composite films exhibit larger orientation change, which is consistent well with the theoretical considerations. The orientation change of Ag@Au NPLs driven by polymer flow could be employed for building pressure sensors indicating the pressure applied on the composite film, if the relation of *Δ(H1/H2)* and the pressure for a batch of duration could be pre-set for a specific kind of composite film.

## Discussion

**Figure 6:**
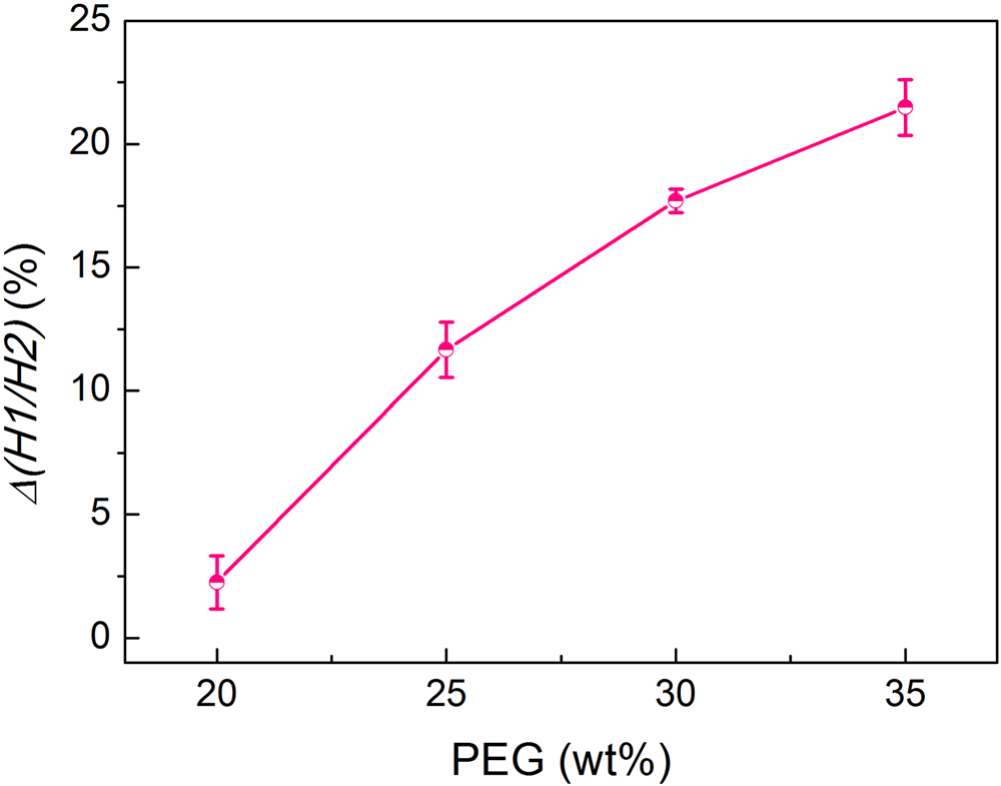
Plot of *Δ(H1/H2)* for composite films with various proportion of PEG experiencing 6 × 10^3^ psi for 2 min.

As demonstrated in Equations (1) and (3), *Δ(H1/H2)* could be influenced by the viscoelasticity of the composite film. By adjusting the ratio between PVP and PEG, we can control the fluidity of polymer to alter the pressure sensitivity of the Ag@Au NPL-polymer composite film and broaden its pressure response range. The deformation of the polymer film became more sensitive to the applied stress with the proportion increase of PEG in the polymer, which can be seen from the variation of extinction spectra of the Ag@Au NPL-polymer composite film. When the pressure is 6 × 10^3^ psi and the duration is 2 min, *Δ(H1/H2)* of the composite film was examined. When the concentration of PEG decreases to 15 wt%, the deformation of composite films is too small that we cannot observe obvious variation of extinction spectra of films. While when the concentration of PEG increases to 40 wt%, the deformation of films changes too fast that we cannot obtain a stable and accurate change of extinction spectra of films. So we only show the results when the concentration of PEG is in the range of 20–35 wt%. As indicated in Fig. 6, when the film containing 20 wt% PEG merely approached a small *Δ(H1/H2)* (2.24%), the rise in the amount of PEG brought larger response to the applied stress with *Δ(H1/H2)* increasing to 11.65%, 17.69%, 21.48% for the composite films with 25 wt%, 30 wt%, 35 wt% of PEG, which demonstrates that the orientation change of Ag@Au NPLs is larger in a more flexible composite film containing a higher proportion of PEG. Thus, by altering the proportion of plasticizer, polymer composite films with various pressure sensitivities for a broad extent of pressure detection can be fabricated. Compared with AuNRs/PVA composite films containing a weak transverse peak, Ag@Au NPLs/PVP composite film prepared in this work has two obvious plasmon peaks, which is favorable for measuring the intensity change used for pressure sensing and could overcome the drawbacks of AuNRs.

In summary, to overcome the drawback of AuNRs with a small transverse peak used for pressure sensing, a novel pressure-responsive film utilizing the orientation change of the LSPR of Ag@Au NPLs has been developed. Under pressure, the directions of Ag@Au NPLs change with the deformation of polymer matrix, as well as the intensity ratio of plasmon peak of AuNPs and in-plane dipolar peak of Ag@Au NPLs. The value of variation in the intensity ratio of these two LSPR peaks is determined by the intensity and duration of external pressure, and it could also be adjusted by the fluidness of the polymer films. Conventional pressure sensors are usually bulky and not favorable for the circumstances for tiny areas or objects with complicated surface. While pressure sensitive film in this work is more adaptable and could be used to record the magnitude and distribution of pressure between two contacting surfaces with complicated topological situations by exporting optical information.

## Methods

### Chemicals

Silver nitrate (AgNO_3_), trisodium citrate dihydrate (TSC), sodium borohydride (NaBH_4_), L-ascorbic acid (AA), polyvinylpyrrolidone (PVP, *M*_w_ = 360 000), polyethylene glycol (PEG, *M*_w_ = 400) were purchased from Sigma-Aldrich. Hydrogen tetrachloroaurate (III) trihydrate (HAuCl_4_·3H_2_O) and PVP(*M*_w_ = 40,000) were purchased from J&K Scientific. Hydrogen peroxide (H_2_O_2_) was purchased from Tianjin Zhiyuan. Sodium hydroxide (NaOH) and sodium sulfite anhydrous (Na_2_SO_3_) were purchased from Sinopharm Chemical. All chemicals were used as received without further treatment.

### Synthesis of Ag nanoplates (NPLs)

AgNPLs were prepared according to the procedure reported by Zhang *et al*. with minor modifications^26^. Typically, aqueous solution of AgNO_3_ (0.03 M, 4 mL), TSC (75 mM, 28.8 mL), and H_2_O_2_ (30 wt%, 3.9 mL) were combined and vigorously stirred at 30 **°**C in air. An aqueous NaBH_4_ solution (0.1 M, 3 mL) was quickly injected into the mixture, producing NPL seeds after 30 min. This solution were centrifuged at 10000 rpm for 12 min and redispersed into 178 mL of deionized water to acquire the seed solution. In a typical seeded growth process, aqueous solution of TSC (75 mM, 1.113 mL) and of AA (0.1 M, 3.338 mL) were rapidly injected into the seed solution under magnetic stirring. A separate solution was prepared by mixing aqueous solution of AgNO_3_ (0.03 M, 4 mL), TSC (75 mM, 2.4 mL) and deionized water (153.6 mL), and then injected into the seed solution dropwise by a syringe pump with the injection rate of 0.5 mL/min. After seeded growth, the final solution was utilized as the stock solution of Ag NPLs.

### Synthesis of Ag@Au NPLs

Ag@Au NPLs were synthesized using a process reported by Gao *et al*.^33^. The growth solution of gold was prepared as follows: aqueous solution of HAuCl_4_ (0.25 M, 40 μL), NaOH (0.2 M, 240 μL), and Na_2_SO_3_ (0.01 M, 3 mL) were dissolved in deionized water (4.72 mL). The solution remained undisturbed for 4~6 h before using. Typically, 40 mL of AgNPLs were centrifuged and redispersed into 2 mL of deionized water. A solution was prepared by mixing aqueous solution of PVP (5 wt%, *M*_w_ = 40,000, 1 mL), NaOH (0.5 M, 0.2 mL), AA (0.5 M, 0.2 mL), Na_2_SO_3_ (0.1 M, 50 μL), deionized water (2.55 mL), and growth solution of gold (4 mL), which was then mixed with AgNPL solution to start the seeded growth. The mixture remained undisturbed at 60 **°**C for 2 h. Finally, Ag@Au NPLs were obtained by centrifugation and redispersed in 0.5 mL of deionized water.

### Synthesis of Au nanoparticles (NPs)

PVP-capped AuNPs were fabricated by a slightly modified Turkevich method^34, 35^. Aqueous solution of HAuCl_4_ (0.02 wt%, 95 mL) was heated to reflux with magnetic stirring, followed by the quick injection of aqueous solution of TSC (1 wt%, 5 mL). The mixed solution was allowed to reflux for 30 min while the color of solution changed from yellow to ruby red, which indicates the formation of citrate-capped AuNPs. The resulting solution was then cooled down to room temperature, mixed with aqueous solution of PVP (12.8 mg/mL, *M*_w_ = 40,000, 4 mL), and gently stirred overnight. After ligand exchange, the PVP-capped AuNPs were centrifuged and redispersed in 5 mL of deionized water.

### Fabrication of Ag@Au NPL-polymer composite films

To obtain 11.1 wt% PVP solution, 10 g PVP (*M*_w_ = 360000) was dissolved in 80 g ethanol under 60 **°**C and mechanical stirring for 6 h till complete dissolution of PVP. The solution was then cooled down to room temperature. Typically, 11.1 wt% PVP solution was mixed with a desired amount of 20 vol% PEG solution, as detailed in Table S1 (total volume of PVP/PEG polymer is fixed). Then the solution was added 94 μL of Ag@Au NPLs and 108 μL of AuNPs to form a homogenous mixture. The mixture was casted into a Teflon mold with circular wells (depth of 25 mm and diameter of 22 mm) and left in room temperature to allow the evaporation of ethanol. Solid films were then peeled off and cut into small discs (diameter of 3 mm).

### Compression tests

The compression tests were performed on the homemade equipment utilized for *in-situ* real-time UV-vis measurement, as seen in Figure S2. Composite films were first loaded into sapphire anvil cell utilized for delivering the external constant force. Then the pressure was applied on the composite film and calculated by dividing the force by the area of sapphire anvil cell (diameter of 3mm) whose area is the same as the initial area of the composite film. The reference of extinction spectra of Ag@Au NPLs and AuNPs in the polymer is the corresponding blank polymer containing the same composition of PVP and PEG but without Ag@Au NPLs or AuNPs.

### Characterization

The morphology of products was observed using a transmission electron microscope (TEM, JEOL JEM 2100). The UV-vis spectra of the composite films were measured by a probe-type Ocean Optics HR2000CG-UV-NIR spectrometer to get the *in-situ* real-time spectra during compression tests. A micrometer was employed to measure the thickness of the composite film.

### DDA simulations

The extinction profiles of AgNPLs with various orientations were calculated by the discrete dipole approximation (DDA) method through software established by Draine and Flatau (DDSCAT 7.3)^30, 40^. DDA is a general method that could calculate the optical properties of noble metal nanoparticles with arbitrary geometry and composition. The metal nanoparticle which is usually called target is replaced by an array of N point dipoles in this approximation. In this work, the target consisted in a triangular prism, with a side width of 90 nm and a thickness of 20 nm (the total thickness of prism is 20 nm and the thickness of shell is 4 nm). We used 8790 dipoles for the target. To obtain the extinction profiles in various orientations, the target was rotated through the angle *θ* which specify the direction of **a**_**1**_ (corresponding to the base plane of AgNPLs) with respect to the incident wave vector **k**. The wave vector was supposed to be along the *x*-axis, while the polarization along both *y*-axis and *z*-axis. The effect of solvent has been accounted for through the refractive index of ambient environment, that is n = 1.333 for water. The dielectric constants of Ag and Au obtained from the reports of Rakić *et al*.^41^ and Johnson *et al*.^42^ were used.

## Electronic supplementary material

Supplementary Information
