# Supplementary material for: Design, Fabrication and Characterization of Pressure-Responsive Films Based on The Orientation Dependence of Plasmonic Properties of Ag@Au Nanoplates
Source: Sci Rep. 2017 May 10;7:1676. doi: 10.1038/s41598-017-01928-5 (PMC5431991; doi:10.1038/s41598-017-01928-5)
Supplement: Supplementary file 1 — Supplementary Information [file 41598_2017_1928_MOESM1_ESM.doc]

*Supplementary Information for*

Design, Fabrication and Characterization of Pressure-Responsive Films Based on The Orientation Dependence of Plasmonic Properties of Ag@Au Nanoplates

Li-Shun Fu^1,2,3^, Wen-Shou Wang^1,2,4^, Cheng-Yan Xu^1^, Yao Li^5^ & Liang Zhen^1^

^1^ School of Materials Science and Engineering, Harbin Institute of Technology, Harbin 150001, China. ^2^ Department of Chemistry, University of California, Riverside, CA 92521, USA. ^3^ Kuang-Chi Institute of Advanced Technology, Shenzhen 518057, China. ^4^ National Engineering Research Center for Colloidal Materials and School of Chemistry and Chemical Engineering, Shandong University, Jinan 250100, China. ^5^ Center for Composite Materials, Harbin Institute of Technology, Harbin 150001, China.

Correspondence and requests for materials should be addressed to W.S.W (wangws@sdu.edu.cn) or L.Z. ([lzhen@hit.edu.cn](mailto:lzhen@hit.edu.cn))


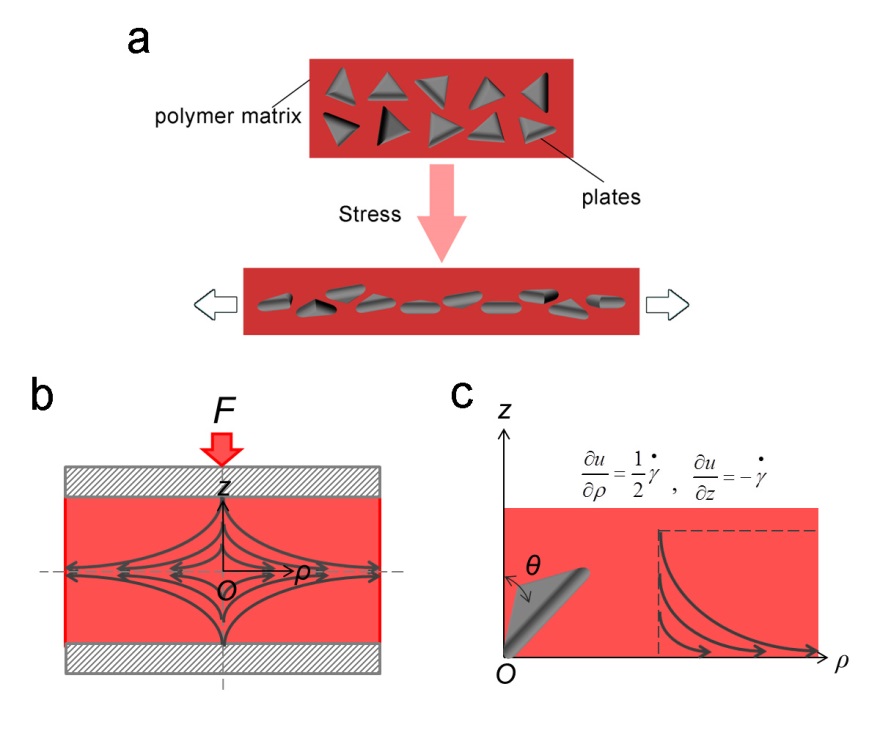


**Figure S1.** (a) Schematic representation of the design of the pressure sensitive film based on orientation variation of LSPR of Ag@Au NPLs. (b) Velocity distribution of the polymer during the deformation of composite film. (c) Coordinate system for the analysis of orientation variation of a single plate-like particle with the aspect ratio *r* in the planar extensional flow.


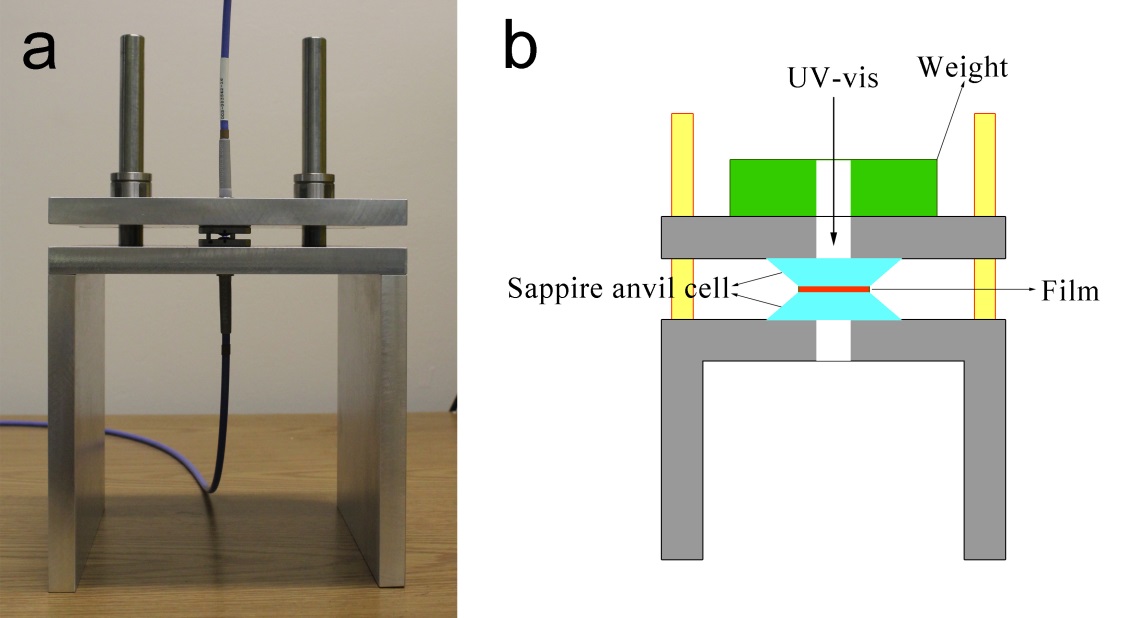


**Figure S2.** Photograph (a) and illustration (b) of homemade device utilized for *in-situ* real-time UV-vis measurement.


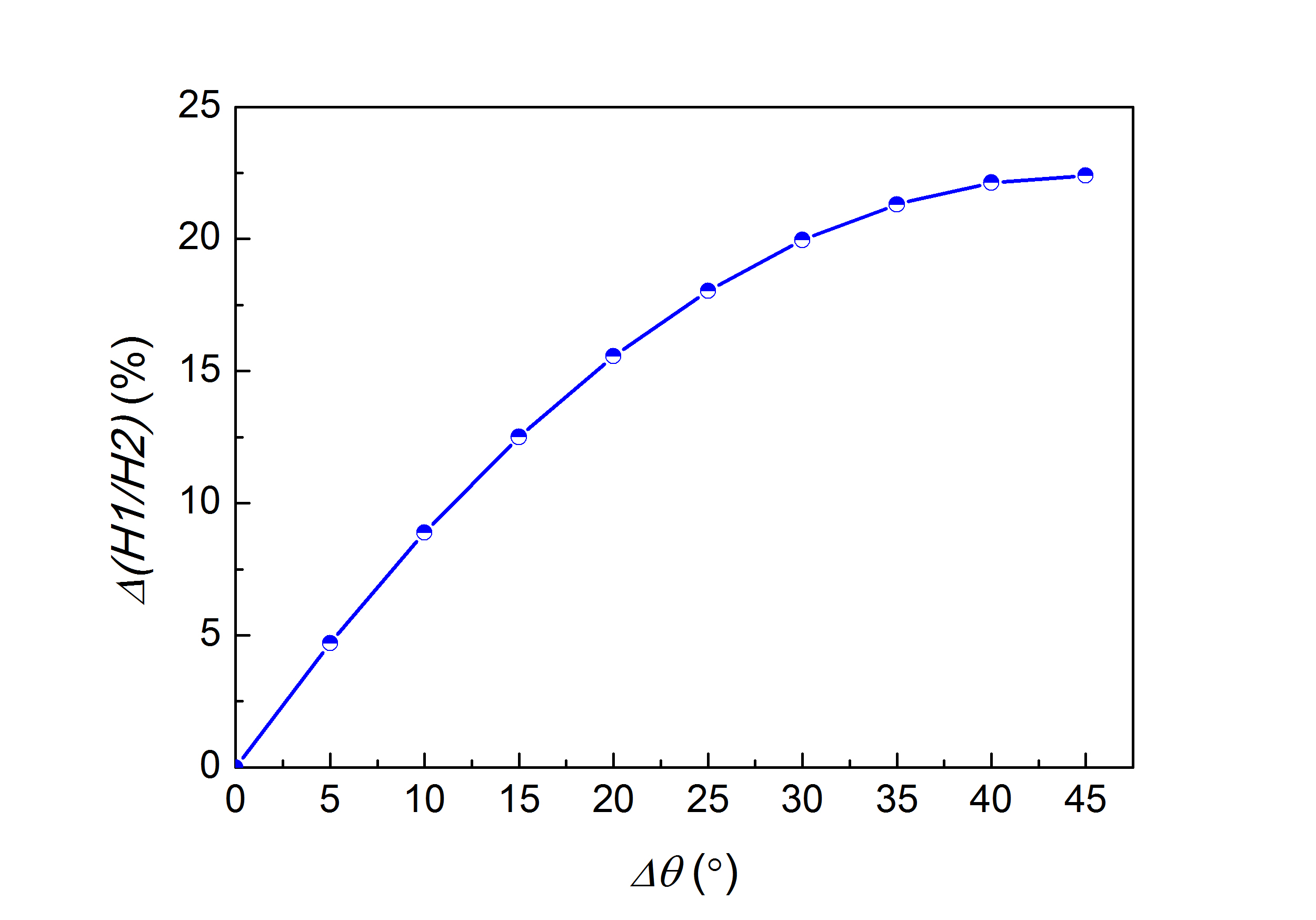


**Figure S3.** Plot of *Δ(H1/H2)* for individual Ag@Au NPLs varying orientation derived from the DDA simulation results. The initial orientation angle of Ag@Au NPLs is set as 45° and rises in a step of 5°.


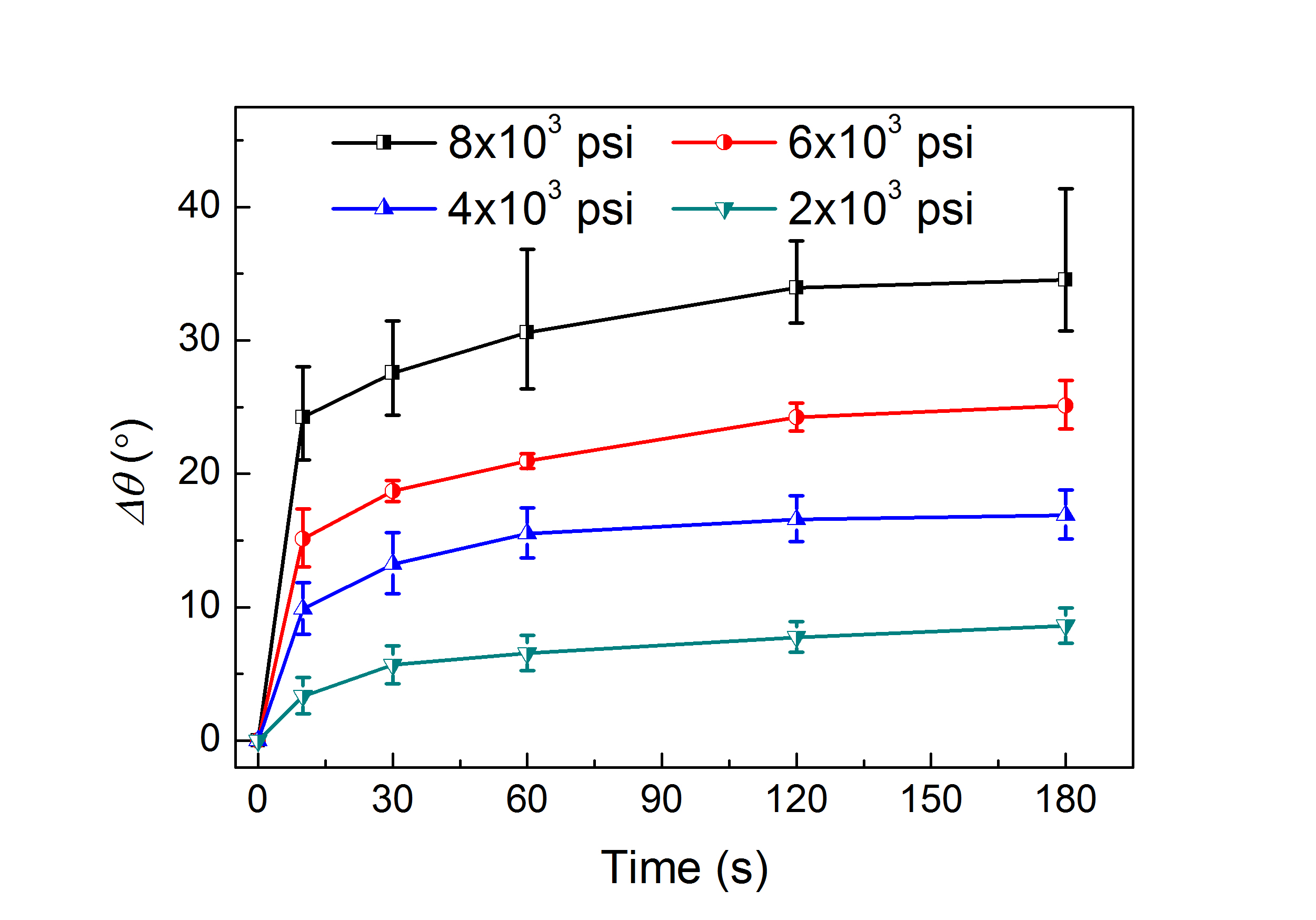


**Figure S4.** Plots of direction change of Ag@Au NPLs in the films that experienced various pressures for a series of duration. The initial angle between the Ag@Au NPLs and incident light is set as 45°.

**Table S1.** Fabrication conditions of Ag@Au NPL-polymer composite films

| Proportion of PEG | 11.1 wt% PVP solution | 20 vol% PEG solution |
| --- | --- | --- |
| 35 wt% | 1.17 g | 310 μL |
| 30 wt% | 1.26 g | 265 μL |
| 25 wt% | 1.35 g | 220 μL |
| 20 wt% | 1.44 g | 180 μL |
